# Taxonomic study of the genus *Neurotettix* Matsumura (Hemiptera, Cicadellidae, Deltocephalinae) with a description of a new species from China

**DOI:** 10.3897/zookeys.82.1158

**Published:** 2011-02-23

**Authors:** Renhuai Dai, Jichun Xing, Zizhong Li

**Affiliations:** Institute of Entomology, Guizhou University; The Provincial Key Laboratory for Agricultural Pest Management of Mountainous Region, Guiyang, Guizhou, P.R. China, 550025

**Keywords:** Hemiptera, morphology, taxonomy, distribution

## Abstract

This paper treats all four known species of the genus Neurotettix Matsumura, 1914 (Hemiptera, Cicadellidae, Deltocephalinae), including one new species: Neurotettix truncatus **sp. n.** from China. A key is given to distinguish all species of the genus, and illustrations of genitalia are provided.

## Introduction

The leafhopper genus Neurotettix, belonging to tribe Athysanini of subfamily Deltocephalinae (Hemiptera: Cicadellidae), was established by Matsumura (1914) for a single species, Neurotettix horishanus Matsumura from Taiwan. Later, Ishihara (1963) redescribed and illustrated this genus. Cai and Shen (1999) described a second species, Neurotettix bifurcatus,from China,and recently, Shen et Dai (2002) describeda third species, Neurotettix flangenus, from China. All the species of this genus are described from China.

Here we described and illustrated a new species from Guizhou Province, China. The type specimens of new species are deposited in the Institute of Entomology, Guizhou University, Guiyang, China (GUGC). The genus Neurotettix now contains four species. A key is given to separate all species.

## Taxonomy

### Neurotettix

Matsumura

Neurotettix Matsumura 1914: 192; Esaki and Ito 1954: 128; Ishihara 1963: 223.

#### Type species:

Neurotettix horishanusMatsumura, 1914

#### Description.

Medium sized leafhoppers, body elongate, vertex triangular and produced. Head including eyes clearly narrower than pronotum. Eyes black, large. Ocelli located on anterior margin of vertex, near eyes. Face with dark, transverse streaks. Frontoclypeus long and narrow. Pronotum longer than vertex, its length of lateral carina 1/3 basal width of eye, anterior margin roundly protruded and posterior margin concave. Scutellum triangular, slightly shorter than pronotum, with transverse suture curved and depressed. Forewings with four apical cells and three subapical cells, apical cells short, anteapical cells with reticulate veins, clavus irregularly reticulated with many extra veins, appendix small.

Male pygofer side with about 10 stout setae, its ventro-posterior margin with a long appendage. Valve triangular. Subgenital plate with many setae in lateral margin. Aedeagus asymmetrical or symmetrical, base robust, aedeagal shaft slender or robust, with or without processes, gonopore apical or subapical. Connective nearly X-shaped. Style slender, elongate.

**Figures 1–15.**
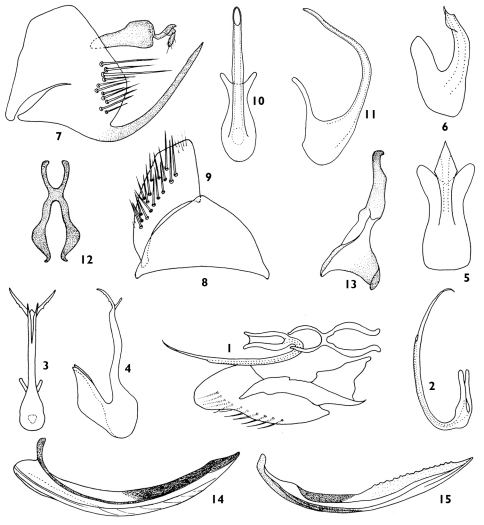
Neurotettix horishanus Matsumura **1** Aedeagus, dorsal view **2** Aedeagus, lateral view (after Ishihara 1963) Neurotettix bifurcatus Cai & Shen **3** Aedeagus, ventral view **4** Aedeagus, lateral view (after Cai and Shen 1999) Neurotettix flangenus Shen & Dai **5** Aedeagus, ventral view **6** Aedeagus, lateral view (after Shen & Dai 2002) Neurotettix truncatus sp. n. **7** Male pygofer side, lateral view **8** Valve, ventral view **9** Subgenital plate, ventral view **10** Aedeagus, ventral view **11** Aedeagus, lateral view **12** Connective **13** Style, dorsal view **14** female 1st valvula, lateral view **15** female 2nd valvula, lateral view.

**Figures 16–19.**
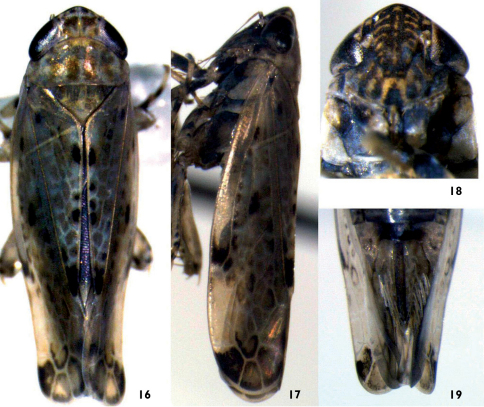
Neurotettix truncatus sp. n. **16** *♂*, dorsal view **17** *♂*, lateral view **18** *♂*, face **19** ♀ abdominal sternum VII, ventral view.

#### Diagnosis.

Neurotettix may be distinguished from other Athysanini by the following combination of features: forewings with apical cells short, anteapical cells with reticulate veins, clavus irregularly reticulated with many extra veins; connective nearly X-shaped.

#### Distribution.

Oriental Region and Palaearctic Region.

#### Disscussion.

The aedeagus is asymmetrical in the type species in original description (Ishihara 1963). But, the aedeagus is symmetrical in the other two species (Neurotettix bifurcatus and Neurotettix flangenus) and the new species Neurotettix truncatus sp. n. According to the external characters and other male genitalia features, we confirmed the other two species and the new species belong to the genus Neurotettix.

#### Key to species (male) of Neurotettix

**Table:** 

1	Aedeagal shaft short and robust (Figs 5, 6)	Neurotettix flangenus Shen & Dai
–	Aedeagal shaft long and slender	2
2	Aedeagal with two paired apical processes (Figs 3, 4)	Neurotettix bifurcatus Cai & Shen
–	Aedeagal without paired apical process	3
3	Gonopore subapical, about one-third from apex (Figs 1, 2)	Neurotettix horishanus Matsumura
–	Gonopore at apex (Figs 10, 11)	Neurotettix truncatus Dai, Xing & Li, sp. n.

### Neurotettix horishanus

Matsumura

Figs 1–2

Neurotettix horishanus Matsumura 1914: 193; Ishihara 1963: 224.

#### Distribution:

China (Taiwan).

### Neurotettix bifurcatus

Cai & Shen

Figs 3–4

Neurotettix bifurcatus Cai and Shen 1999: 41–42.

#### Distribution:

China (Henan).

### Neurotettix flangenus

Shen & Dai

Figs 5–6

Neurotettix flangenus Shen and Dai 2002: 89–91.

#### Distribution:

China (Hunan).

### Neurotettix truncatus

Dai, Xing & Li sp. n.

urn:lsid:zoobank.org:act:41C31A94-D0A4-4E56-96CC-BBC3D49AB654

Figs 7 19 

#### Description.

Body yellow-brown, vertex with black spots along anterior margin, and with two irregular orange markings behind the spots, eyes dark-brown (Fig. 16). Face brown, transverse streaks and a longitudinal band yellow, anteclypeus yellow with apex dark-brown (Fig.18). Pronotum and scutellum yellowish-brown. Forewings yellowish-brown, with irregular fuscous markings, viens yellowish-white, apical part of forewings pale brown (Fig. 17). Female abdominal genital segment pale brown (Fig. 19).

External features as in generic description.

*Male genitalia*. Pygofer side short, with eleven stout setae along dorso-caudal margin, its ventro-posterior margin with a long appendage (Fig. 7). Valve triangle (Fig. 8). Subgenital plate short and broad, with many setae in lateral margin, distally truncate, with 2 to 3 irregular rows of setae from lateral margin to middle of subgenital plate (Fig. 9). Aedeagus symmetrical, base robust, aedeagal shaft slender without processes, gonopore apical (Figs 10, 11). Connective nearly X-shaped, its arms longer than stem (Fig. 12). Style slender, elongate, with apex of apophysis curved laterally (Fig. 13).

Female seventh sternum concaved medially on posterior margin. First valvula of ovipositor sculpture irregularly (Fig. 14), second valvula with teeth, tapered toward apex in lateral view (Fig. 15).

#### Measurement.

Length (including tegmen): *♂*4.8–5.3mm, *♀* 4.9–5.3mm.

#### Type Material.

Holotype *♂*, China: Guizhou Prov., Kuankuoshui, 16 August 2010, coll. Jichun Xing (GUGC). Paratypes: 1*♂*2♀♀, Guizhou Prov., Kuankuoshui, 12 August 2010, coll. Renhuai Dai (GUGC), 1*♂*1♀, China: Hubei Prov., Lichuan city, Pingba, 1 August 2010, coll. Junqiang Ni (GUGC).

#### Host.

Bamboo.

#### Remarks.

This species is similar to Neurotettix horishanus Matsumura, but can be distinguished from the latter by the symmetrical aedeagus and gonopore at apex, subgenital plate distally truncate and style apex curved.

#### Etymology.

The new species name is derived from the Latin words “*truncatus*”, indicating that the subgenital plate distally truncate.

## Supplementary Material

XML Treatment for Neurotettix

XML Treatment for Neurotettix horishanus

XML Treatment for Neurotettix bifurcatus

XML Treatment for Neurotettix flangenus

XML Treatment for Neurotettix truncatus
